# Pathogenicity of *Trichobilharzia* spp. for Vertebrates

**DOI:** 10.1155/2012/761968

**Published:** 2012-10-23

**Authors:** Lichtenbergová Lucie, Horák Petr

**Affiliations:** Department of Parasitology, Faculty of Science, Charles University in Prague, Viničná 7, 128 44 Prague 2, Czech Republic

## Abstract

Bird schistosomes, besides being responsible for bird schistosomiasis, are known as causative agents of cercarial dermatitis. Cercarial dermatitis develops after repeated contact with cercariae, mainly of the genus *Trichobilharzia*, and was described as a type I, immediate hypersensitivity response, followed by a late phase reaction. The immune response is Th2 polarized. Primary infection leads to an inflammatory reaction that is insufficient to eliminate the schistosomes and schistosomula may continue its migration through the body of avian as well as mammalian hosts. However, reinfections of experimental mice revealed an immune reaction leading to destruction of the majority of schistosomula in the skin. Infection with the nasal schistosome *Trichobilharzia regenti* probably represents a higher health risk than infections with visceral schistosomes. After the skin penetration by the cercariae, parasites migrate via the peripheral nerves, spinal cord to the brain, and terminate their life cycle in the nasal mucosa of waterfowl where they lay eggs. *T. regenti* can also get over skin barrier and migrate to CNS of experimental mice. During heavy infections, neuroinfections of both birds and mammals lead to the development of a cellular immune response and axonal damage in the vicinity of the schistosomulum. Such infections are manifest by neuromotor disorders.

## 1. Introduction

Despite their worldwide distribution, avian schistosomes were neglected by parasitologists who assumed that they have no or minor pathogenic impact on birds or mammals, including humans. Nowadays, many studies focus on these parasites since it has been recognized that they can be severe pathogens of birds. Moreover, their larval stages (cercariae) frequently infect humans and cause cercarial dermatitis. The most reported agents of swimmer's itch are cercariae of the genus *Trichobilharzia *[1].

Human infections by bird schistosomes are associated mostly with the development of cercarial dermatitis (swimmer's itch), an allergic skin response, which develops after repeated contact with cercariae penetrating into the skin. For a long time, it was assumed that the reaction eliminated the majority of the schistosomes that had penetrated into the skin. However, the studies on mice infected experimentally with bird schistosomes showed that soon after the penetration, the cercariae transform to schistosomula. Under certain circumstances, these schistosomula are able to resist host immune response, escape from the skin, and migrate further to target organs [2, 3]. In mammals, bird schistosomes can survive for several days or weeks, but they never mature [4, 5]. The exact reason why bird schistosomes die in mammalian hosts has not been known until the present. 

Studies on bird schistosomes disclosed a new species—*Trichobilharzia regenti* [4]— with unusual behavior in compatible as well, noncompatible hosts. In comparison to the majority of bird schistosome species living in the blood system of visceral organs, mature *T. regenti* occur in the nasals of their definitive host where they lay eggs. Migration of the worms from the skin to the nasals is via the spinal cord and brain [4]. Experimental infections of mice showed that *T. regenti* schistosomula can evade attack by immune cells in the skin of mammalian hosts allowing them to migrate further through the central nervous system (CNS) where immature worms die after several days [5, 6]. Migration of the parasites through CNS of both bird and mammalian hosts causes severe tissue injuries [6, 7] that can result in leg paralysis, balance, and orientation disorders and even host death [4, 7].

Nowadays, mainly two species of bird schistosomes, *T. szidati* and *T. regenti*, are studied under laboratory conditions with regard to their development, physiology (including enzymes participating in host tissue degradation and digestion), immunomodulation of the host immune response, and pathogenicity towards natural and accidental hosts. In field studies, the emphasis is on the study of species spectrum, inter- and intraspecific variability, and prevalence of bird schistosomes (see, e.g., Brant and Loker [8], Jouet et al. [9], and Korsunenko et al. [10]; see also review by Horák and Kolářová [1]). With regard to *T*. *szidati* and *T*. *regenti*, their occurrence has been reported from several countries. In particular, *T*. *regenti* cercariae have been found in freshwater ponds for example, in Russia [11] and cercariae of *T*. *szidati *in Russia, Belorussia [11], Germany [12] and France [13]. Several findings of *T*. *szidati* infections in birds were reported, for example, from France [9], Poland, and Czech Republic [14]. Infections of birds with *T*. *regenti* were detected for example, in Iceland [15] and in France, where the prevalence on three studied localities reached 40% [9]. Based on findings of Rudolfová et al. [14, 16], prevalence of *T*. *regenti *infection of waterfowl was 14% in Czech Republic (one studied locality) [16] and 22% in Gdansk area in Poland [14]. Although *T*. *szidati* cercariae are mostly distributed throughout Europe, there is a report of their occurrence in snails collected from Michigan and Montana in the United States [8].

The main aim of our review is to summarize the present knowledge of the pathogenesis of bird schistosomiasis and the immune reactions to bird schistosomes presence in avian and mammalian hosts, with a special emphasis on *T*. *regenti*. The neurotropic species *T*. *regenti*, due to its unusual mode of migration and potential pathogenic impact on avian as well as mammalian hosts, deserves more attention. Therefore, a major part of this review is dedicated to this species of schistosome.

## 2. Skin Infection

After leaving the snail intermediate hosts, bird schistosome cercariae have a tendency to cling to the water surface and wait for their definitive host. They react to shadow stimuli and start to swim with a negative phototactic orientation from the water surface toward the definitive host [17]. Except for physical stimuli such as shadow, water turbulence, and warmth, cercariae respond to host chemical cues like duck-foot skin lipids—cholesterol and ceramides [17, 18]. 

Once attached to the host skin, cercariae creep on the skin and search for a suitable penetration site [19]. In contrast to human schistosomes, such as* Schistosoma mansoni*, which penetrate smooth skin, bird schistosome cercariae prefer skin wrinkles and hair follicles for penetration [20]. Studies on cercarial behavior of *S*. *mansoni* and *T. szidati* revealed differences in the speed of migration through the host skin. For example, cercariae of *T*. *szidati* invade human skin more efficiently than *S. mansoni* such that they are able to locate entry sites and penetrate through the skin more rapidly than *S. mansoni* [20]. 

Skin penetration by cercariae is stimulated by fatty acids [19]. According to the study of Haas and Haeberlein [20], *T. szidati *cercariae respond to linolenic acid with higher sensitivity if compared to *S. mansoni*. This feature seems to represent an adaptation to invade duck skin that has a lower content of free fatty acids compared to human skin [20]. Therefore, human skin with higher amount of surface lipids is likely more attractive to bird schistosome cercariae than duck skin [19].

Penetration through the skin is facilitated by a number of proteolytic enzymes, which are released from cercarial circum- and postacetabular glands immediately after attaching to the host skin. In the case of bird schistosomes, glandular secretion is stimulated mainly by fatty acids, ceramides, and cholesterol [20]. Cercarial glands fill about one-third of the cercarial body [21] and contain many of the potentially antigenic proteins. The most important penetration enzyme of *S. mansoni* is probably a serine protease, elastase [22]. Nevertheless, Mikeš et al. [23] and Kašný et al. [24] did not find any elastase activity in the secretions of *T. szidati *and *T. regenti *cercariae, and it was not found in the congener *S. japonicum *[25]. However, cathepsin B-like activity was detected in the aforementioned species. This enzyme from cercarial penetration glands is considered to be the main component in the cercarial penetration process [23–25]. The same types of enzymes could be the reason for similar penetration speed of *S. japonicum *and *Trichobilharzia* cercariae [20]. In addition, six isoforms of cathepsin B1 (TrCB1.1–TrCB1.6) and cathepsin B2 (TrCB2) were identified in an extract of migrating *T. regenti* schistosomula [26, 27]. Two isoforms, TrCB1.1 and TrCB1.4, degrade myelin basic protein, but do not efficiently cleave hemoglobin [26]. The recombinant form of TrCB2 is able to cleave protein components of the skin (keratin, collagen, and elastin) as well as nervous tissue (myelin basic protein), but has negligible activity towards hemoglobin [27]. The enzyme could, therefore, serve as a tool for migration through the host skin and subsequently through the nervous tissue. 

Host fatty acids seem to stimulate not only the penetration of cercaria through the host skin, but also transformation of their tegument as a part of parasite immune evasion [19]. Penetration of the cercariae into the host skin is accompanied by cercaria/schistosomulum transformation with reconstruction of tegumental surface. Transformation starts with loss of tail, a process supported by a sphincter muscle in cercarial hindbody [19], then the cercariae shed the glycocalyx and start to form a surface double membrane. Creation of a new surface is accompanied by the disappearance of lectin and antibody targets on the surface of the schistosomula [28].

In the skin of the bird hosts, schistosomula move through the skin towards deeper layers and, therefore, require information for orientation. Studies on visceral schistosomes invading humans, *S*. *mansoni*, and birds, *T. ocellata*, showed that schistosomula use negative photo orientation to move away from light source [29]. The other stimulus involved in navigation of the visceral schistosomula is represented by the concentration gradient of chemicals, such as D-glucose and L-arginine [30]. Unfortunately, data about orientation of the nasal species *T. regenti* are not complete, but there is an indication that the stimuli differ from those used by visceral species (unpublished).

## 3. Cercarial Dermatitis

In humans, the skin infection is a result of the development of an inflammatory reaction known as swimmer's itch or cercarial dermatitis. Cercarial dermatitis can occur after contact with water containing cercariae from snails infected by bird schistosomes. Chances of getting cercarial dermatitis increase with repeated exposures to the parasite. Higher incidence of the infection is connected with bathing in shallow water, which is the preferred habitat for water snails and, therefore, a place where cercariae accumulate [31].

Penetration of cercariae into the skin may result in an immediate prickling sensation that lasts for about 1 hour [32]. Severity and intensity of cercarial dermatitis depend on various factors including the number and duration of exposures to the cercariae, and host immune status, that is, history of cercarial dermatitis, and individual susceptibility to the infection [32]. After a primary infection, the skin reaction is unapparent or mild with small and transient macules or maculopapules, which develop after 5–14 days [32]. The most pronounced disease occurs after repeated exposures that result in a strong inflammatory reaction against the parasites [33]. The skin disease manifests by maculo-papulovesicular eruptions accompanied by intense itching and, occasionally, by erythema, fever, local lymph node swelling, oedema. Massive infections may also cause nausea and diarrhea (for a review see Horák et al. [34]). Skin lesions develop only on those parts of body where there was cercarial penetration [35].

Diagnosis of cercarial dermatitis is based on anamnesis and clinical findings [36]. Some work has been done using serological tests for confirmation of the diagnosis [37]. Nevertheless, immunological tests are not routinely available and laboratory confirmation of causative agents of the dermatitis remains difficult. 

## 4. Skin Immune Response

Clinical pattern of cercarial dermatitis is linked with histopathological reactions to the infection. In the case of the human schistosome, *Schistosoma mansoni*, infections in naive mice led to a mild skin response with contribution of neutrophils and mononuclear cells. In contrast, a more severe cellular reaction developed in the mouse skin after repeated infections with *S*. *mansoni* [38]. Similarly as for human schistosomes, infections of mice with the bird schistosomes *T. szidati* (*T. ocellata*) and* T. regenti *initiate the development of a skin immune response [2, 39]. Primary mouse infection with *T. regenti *initiates an acute inflammation with oedema, vasodilatation, and tissue infiltration by neutrophils, macrophages, mast cells, and MHC II antigen presenting cells (APCs), and a weak infiltration by CD4^+^ lymphocytes; repeated infections cause substantially elevated infiltration of all cells mentioned above [2, 6]. *Trichobilharzia regenti* primoinfection leads to the development of an inflammatory reaction in the murine skin within 1–6 h after exposure [2]. Detection of cytokine production by *in vitro* cultured biopsies of pinnae (later skin biopsies) obtained from primoinfected mice revealed that the inflammation is accompanied by a transient release of acute phase cytokines (IL-1*β* and IL-6) and increasing amounts of IL-12 [2]. Increased production of IL-12 in the skin correlated with higher Th1-associated IFN-*γ* production by cells from skin-draining lymph nodes [2]. Similarly, the study of Hogg et al. [40] using *S. mansoni* illustrates rapid host immune response to parasite penetration with production of acute phase cytokines, such as IL-1*β* and IL-6 which were detected in the supernatants of skin biopsies from wild-type mice. Both IL-1*β* and IL-6 promote Th17-cell differentiation [41], therefore implying that primary mouse infection with human as well as avian schistosomes induces a Th17 polarized response.

Skin immune response to challenge infections leads to capture and elimination of the majority of schistosomula in the skin [2]. In the early phase after re-infection with *T. regenti* cercariae, infiltration of mouse skin with inflammatory cells (high density of granulocytes and neutrophils, abundant MHC II APCs, macrophages and CD4^+^ lymphocytes) was also accompanied by oedema caused by local vascular permeability that was initiated by histamine produced by activated mast cells and basophils [2]. Degranulation of mast cells and basophils with release of histamine and IL-4 is realized after binding of IgE-antigen complex via high affinity receptors Fc*ε*RI on the cell surface [42, 43]. Histamine has been described previously as a potent effector of Th1 and Th2 responses as well as immunoglobulin synthesis [44, 45]. Repeated infections with *T. regenti* evoke dominant production of Th2-type cytokines, and the first and most abundant cytokine detected in supernatants of skin biopsies from mice after repeated infections is IL-6 [2], which can initiate Th2-type polarization via induction of IL-4 [46]. Within 1 hour after the penetration by *T*. *regenti* cercariae a massive upregulation of IL-4 and IL-10 can be observed in the skin biopsies, and the level of these cytokines declines after 48 h. This upregulation during *T*. *regenti *infection is accompanied by release of histamine and proliferation of mast cells [2]. Production of histamine and IL-4 detected in skin biopsies immediately after the last infection of re-infected mice was realized via IgE-dependent mast cell degranulation [2]. IL-4 plays also a crucial role in the development of Th2-type immune responses to *S. mansoni* antigens and regulation of immunoglobulin isotype switch to IgE [47]. CD4^+^ cells, numbers of which significantly increase in the skin after challenge infections, are potential sources of IL-4 associated with Th2 response [2].

Like mast cells, basophils possess high-affinity IgE surface receptors (Fc*ε*RI) that, after antigen-specific cross-link, induce production and release of mediators such as histamine and IL-4 [48]. *In vitro *stimulation of basophils obtained from healthy (nonsensitized) humans by homogenate of cercariae and excretory/secretory (E/S) products of *T*. *regenti *cercariae revealed that these antigens induce basophil degranulation and release of IL-4 [49]. Antigens stimulated basophil release of IL-4 in a dose-dependent manner, and antigens from E/S products were more potent inducers of IL-4 release than cercarial homogenate [49]. Elevated levels of skin mast cells [2] and high titers of serum IgE in mice infected repeatedly with *T. regenti *[49] showed that the cells of mast cell/basophil lineage play an important role in development of Th2 responses during *Trichobilharzia *infections.

## 5. Antibody Response and Antigens of Bird Schistosomes

Domination of Th-2 polarization of the immune response after repeated *T. regenti* infections was confirmed by measurement of antigen-specific antibody levels. Similarly as in the study of Kouřilová et al. [2], the increase of Th2-associated antigen-specific IgG1 and total serum IgE antibodies with concurrent decline of Th1-associated IgG2b antibody was demonstrated in sera of mice repeatedly infected with *T. regenti* [49].

During *T. regenti* primary infection, IgM response against glycan structures of the cercariae and their excretory/secretory (E/S) products was observed [49]. This indicates that early antibody response is directed against components of highly antigenic cercarial glycocalyx as well as against glycoproteins contained in E/S products of circum- and postacetabular glands of cercariae [49]. The glycocalyx of *T*. *regenti *cercariae was described as the most antigenic structure and its remnants were still present on 1-day old schistosomula transformed *in vitro *[50], therefore IgM antibodies could recognize components of the glycocalyx during primary infection.

After penetration into the host skin, cercariae transform to schistosomula by shedding their tails, releasing E/S products, and rebuilding their surface [51]. The dominant part of the cercarial surface is represented by glycocalyx, which is likely the main component responsible for complement activation [52]. Cercarial surface of human and bird schistosomes is recognized by antibodies from humans and mice infected with *T*. *regenti*, *T*. *szidati*, and *S*. *mansoni* [53]. In the skin, parasites need to avoid destruction by the host immune system, thus the transformation of cercaria to schistosomulum is accompanied by the decrease of surface saccharides and antigen epitopes which could be recognized by lectins and antibodies, respectively [28]. Although a substantial part of glycocalyx is removed by the cercariae during penetration [54], some of glycocalyx components remain associated with surface of the schistosomula for some time after transformation [54, 55]. Therefore, not only the cercarial surface but also the surface of the early *in vitro *transformed (5 h) schistosomula was strongly recognized by IgG and total Ig antibodies from mice repeatedly infected by *T*. *szidati *[28]. E/S products of human as well as bird schistosome cercariae, mainly the products released by transforming larvae, are rich in components of glycocalyx and secretions of circum- and post-acetabular glands [23, 54].

Immunohistochemical staining of ultrathin sections of particular developmental stages revealed variable distribution of antigens recognized by IgG antibodies from sera of mice re-infected with *T*. *regenti *[50]. Except for antibody binding to the surface of cercariae and 1-day-old schistosomula, a positive reaction with spherical bodies located in subtegumental cells of cercariae and early schistosomula was recorded [50]. In schistosomula, spherical bodies represented the most reactive structure. Further development of the parasite was accompanied with loss of immunoreactivity. In adult worms, the antibodies recognized the surface and subtegumental cells, but with lower intensity if compared to the larval stages [50].

Spherical bodies primary located within subtegumental cells were transferred via cytoplasmic bridges to the surface of schistosomula. As in human schistosomes [56], these bodies probably release their content on surface of the tegument where the antigenic molecules could be recognized by the host immune system [50]. However, composition of the spherical body content is not known till present. 

Nevertheless, based on Western blot analysis of cercarial homogenate probed with sera from re-infected mice, several antigens (14.7, 17, 28, 34, and 50 kDa) were identified by IgG and IgE antibodies. Antigens of 34 and 50 kDa were also recognized in cercarial gland secretions [49]. A precise identification of the 34 kDa molecule which is regarded as a major immunogen is in progress.

## 6. CNS Infection

The bird schistosome *T*. *regenti *exhibits an unusual mode of behavior. After successful escape from the host skin, schistosomula migrate further to the CNS of both specific avian and accidental mammalian hosts [5, 7]. CNS represents an obligatory part on the migration pathway of *T*. *regenti *to the nasal mucosa [4]. In specific bird hosts, schistosomula grow and mature during the migration, and their development is completed in the nasal area of the host [4]. In accidental mammalian hosts, the parasite is not able to complete its development and dies as an immature schistosomula in the spinal cord or brain [4].

**Figure 1:**
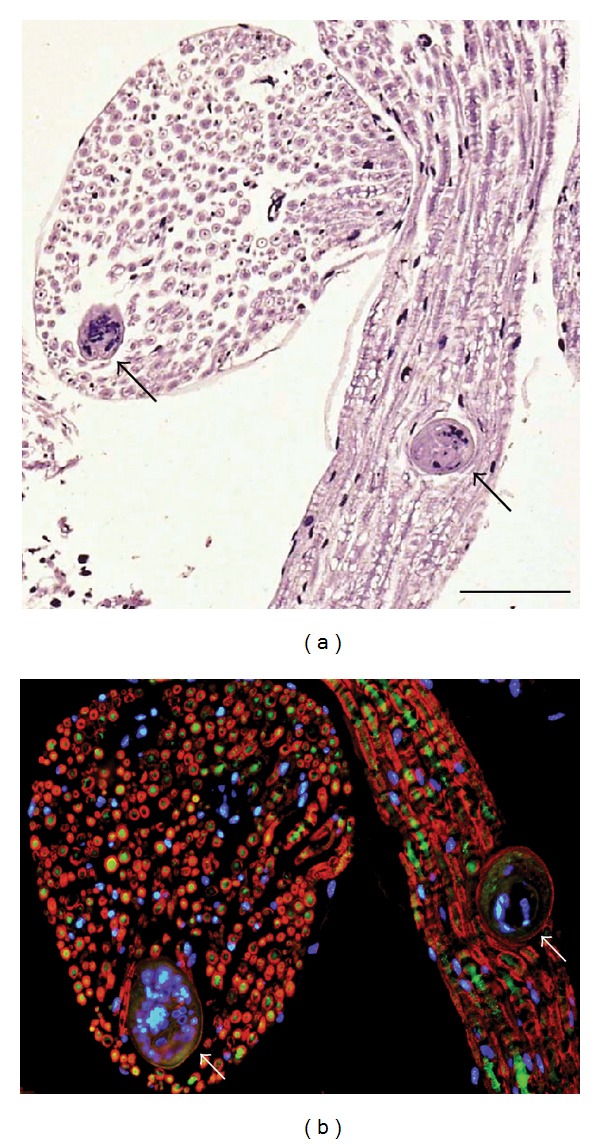
*T. regenti* schistosomula (arrows) migrating inside the peripheral nerve fascicles of an experimentally infected mouse: (a) haematoxylin and eosin staining, scale bar 100 *μ*m; (b) myelin sheets visualized by binding of anti-MBP (myelin basic protein) antibody and secondary anti-rabbit IgG-Cy3 (red), neurofilaments stained by anti-SMI-32 (neurofilament H nonphosphorylated) and secondary anti-mouse IgG-Alexa Fluor (green), cell nuclei stained by DAPI (4′,6-diamidino-2-phenylindole) (blue).

Soon after penetration of cercariae into the skin of specific as well as accidental definitive hosts, schistosomula locate peripheral nerves, enter nerve fascicles (Figure 1) or *epineurium*, and reach the spinal cord via spinal roots [5, 57]. Schistosomula appear in the spinal cord from day 2 post infection (p.i.); intact parasites can be detected in the duck spinal cord even 23 days p.i. [7], and 21–24 days p.i. in the spinal cord of mice [5]. Then, schistosomula migrate to the brain where the parasites occur from day 12 p.i. to day 18 p.i. in the case of infected ducks, and from 3 days p.i. to 24 days p.i. in the case of infected mice [5]. At the beginning of the brain infection, schistosomula are located mainly in *medulla oblongata* and further in subarachnoidal area of *cerebellum* [5, 57]. Other localization in the brain is mostly restricted to the subarachnoidal space and the area of the fourth ventricle [57, 58].

Migration through the host CNS requires parasites adaptations to this environment. Light-brown-pigmented granules in the intestine of *T*. *regenti* schistosomula [59], which exhibit immunoreactivity with antibodies against components of CNS [57], proved that schistosomula utilize host nervous tissue for nutrition during their migration via CNS. For this purpose the parasite should have proteases capable of cleaving components of the nervous tissue. Until the present only cathepsins B1 and B2 with the capability to degrade myelin basic protein [26, 27] were identified as candidate molecules.

Except localization in CNS that is typical for this species, schistosomula were accidentally observed in the lungs of specific avian [4] as well as non-compatible mammalian hosts [5, 57]. Moreover, schistosomula were also found in skin capillaries [53]. However,* in vitro *tests of blood vessel attractiveness did not show any positive results [5]. Therefore, it seems that schistosomulum presence in the lungs represents an ectopic localization of the parasite in heavily infected experimental animals [57]. 

## 7. Immune Response and Pathology in the CNS

Presence of the parasites in CNS initiates a cellular response. In the spinal cord of ducks 23 days p.i., the parasites were located mostly in meninges of thoracic and synsacral spinal cord and in the white and gray matter of synsacral spinal cord [7]. The surroundings of the worms were infiltrated with eosinophils, heterophils, and less frequently with plasma cells, histiocytes, lymphocytes, and macrophages [7]. Despite dense infiltration with the immune cells, the parasites were not destroyed by host immune response [7]. The parasites located in brain, predominantly in meninges, were surrounded by macrophages and endothelial cells [7]. At present, details about the immune response in the infected ducks are still missing.

More studies have been done on mice as a model of non-compatible host. Early infections (3 days p.i.) of the mouse spinal cord did not initiate any inflammatory reaction or damage to the nervous tissue; only focal oedema was observed [6, 57]. Signs of the infection were observed 6 or 7 days p.i., when the vicinity of schistosomula was infiltrated with granulocytes, predominantly neutrophils, activated microglia, macrophages and, less frequently, CD3^+^ lymphocytes [6, 57]. It seems that microglia and macrophages, are responsible for destruction of schistosomula in the mouse CNS [57]. Similarly, microglia are the most important cells in prevention against *Toxoplasma gondii* tachyzoite proliferation in the brain [60]. The presence of *T*. *regenti *schistosomula in the nervous tissue triggered activation and proliferation of astrocytes, which were detected in the tracks after migrating schistosomula [57]. Formation of glial scars by astrocytic processes around inflammatory infiltrates was also observed in the brain of human patients infected with *Taenia solium* metacestodes [61]. The presence of the metacestodes was accompanied by astrocytic activation and increased expression of glial fibrillary acidic protein (GFAP) [61]. Similar activation of astrocytes was detected in the brain of *Toxocara*-infected mice [62]. Progress of *T*.* regenti* infection led to the formation of inflammatory lesions surrounding, destroyed schistosomula in the white matter of the spinal cord. Lesions were formed by microglia, macrophages, eosinophils, neutrophils and CD3^+^ lymphocytes, and damaged axons were detected in the tissue surrounding the lesions. On the other hand, presence of the parasites outside of parenchyma, in subarachnoidal space of the spinal cord and brain, and in the cavity of the 4th ventricle of the brain, did not initiate any heavy inflammation, nor pathological changes in the surrounding tissue. This implies that schistosomula are able to prolong their survival while they migrate outside of parenchyma [57]. Nevertheless, progression of the infection inevitably led to elimination of the parasite. Most schistosomula were destroyed on day 21 p.i. [57]. Challenge infections initiated development of a strong immune response leading to rapid destruction and elimination of schistosomula. No intact worms, only parasite remnants surrounded by inflammatory foci, were observed in CNS 3 days after the fourth infection [6, 57].

Infections of immunodeficient mice (SCID strain) revealed that deficiency in T- and B-cell production led to development of mild immune reaction, which is not sufficient for elimination of the parasite [6, 57]. Even challenge infections did not induce proper immune reactions leading to the destruction of all worms. However, the tissue in the vicinity of intact schistosomula was infiltrated with inflammatory cells, and also axonal damage as a result of schistosomulum migration was observed [57]. A higher number of schistosomula in CNS and prolonged survival of worms indicate the importance of lymphocytes in the destruction of schistosomula [57]. Lymphocyte infiltration in CNS was also demonstrated for other disorders, including neurocysticercosis [63], cerebral malaria [64], and toxoplasmic encephalitis [65]. It was observed that T cells potentiated an immune defense against *Mesocestoides corti *infection of mice by indirect activation of other immune cells (i.e., macrophages) and resident CNS cells (i.e., microglia, astrocytes) [63].

The increased number of *T*. *regenti* schistosomula migrating through the nervous tissue of immunodeficient mice caused axonal damage and activation and proliferation of astrocytes. No significant differences in the number of damaged axons around the inflammatory lesions and in the vicinity of schistosomula were observed in immunocompetent and immunodeficient mice. Therefore, axonal injury was probably caused mechanically by migrating schistosomula and not by the host immune defense [57]. These injuries of the spinal cord resulted in partial hind leg paralysis [57] or even death (unpublished) of infected SCID mice.

## 8. Terminal Phase of the Infection

The exact migratory route from the brain to the site of final location in the nasals is not described. Two hypotheses have been published. Based on the presence of worms in *bulbus olfactorius* [5, 7] a hypothesis about migration via cranial nerves was formulated. Nevertheless, investigation of cranial nerves, *n. olfactorius* and *n. opticus*, did not reveal any parasites or lesions; therefore this hypothesis was rejected [58]. Histological studies showed an extravascular localization of the parasite in subarachnoidal space; several worms were also located intravascularly [7, 58]. The intestine of the worms in meninges contained a dark-brown pigment, probably hematin produced by hemoglobin digestion [58, 59]. These findings support the theory that *T. regenti *probably migrates from the meninges to the nasal cavity via blood vessels [58], but this hypothesis needs to be validated.

The first appearance of parasites in the nasal mucosa was noted 13 days p.i. [4]. Intact live worms were located intravascularly or extravascularly [66] in the connective tissue between cartilage of turbinate and glandular epithelium of the nasal mucosa until 24 days p.i. [58]. The tissue around the adult worms was infiltrated with various inflammatory cells [7]. Fifteen days p.i., immature eggs were detected extravascularly in connective tissue close to cartilage. The highest number of eggs appeared 22 days p.i. and they were distributed all over the nasal mucosa [58]. The eggs with fully developed miracidia were surrounded by a focal dense mass of eosinophils, heterophils, histiocytes, and multinucleated giant cells [7]. Miracidia hatch directly in the nasal tissue and leave the nasal cavity (bill) when the duck submerges its bill in water to feed or drink [34]. Swimming miracidia then actively search for their snail host and a new life cycle of *T*.* regenti* begin.

## 9. Conclusion

Avian schistosomes, in comparison to the extensive studies on human schistosomes, are neglected. The most studied species of avian schistosomes belong to the genus *Trichobilharzia*. Their life cycle is connected to an aquatic environment and they use waterfowl as definitive hosts. Adult worms of the genus *Trichobilharzia* inhabit either visceral or nasal areas of their bird hosts. Visceral species represent the majority of avian schistosomes, whereas nasal species form a small group. *Trichobilharzia regenti* is the only nasal species whose life cycle is described. After penetration into the skin, *T*. *regenti* is able to invade peripheral nerves and continue via CNS to the nasal cavity of birds where adult females lay eggs. Such neuroinfections of birds can result in transient or permanent neuromotor disorders, especially during heavy infections.

Experimental studies on mice revealed that bird schistosomes possess an ability to penetrate into the body of mammals. In case of nonsensitized or immunodeficient mice, the parasites are not eliminated in the skin by the host immune system and can migrate further through the host body; the parasites never mature in mammalian host and die after a few days/weeks after infection. In case of infections with *T*. *regenti*, schistosomula reach the spinal cord and brain of the experimental mice. Migrating schistosomula cause axonal damage in the mouse nervous tissue and initiate development of inflammatory reaction. Neuroinfections, mainly in case of immunodeficient animals, are manifest by hind leg paralysis and even death of the heavily infected host.

Under natural conditions, cercariae of bird schistosomes are attracted not only by the duck-foot skin, but they also positively react to human skin stimuli. In the human skin, cercariae cause an allergic disease known as cercarial dermatitis. It develops after repeated contact with cercariae as a response of the host immune system to antigens of penetrating parasites, and leads to the destruction of parasites in the skin. 

Based on recent reports of bird schistosomes and outbreaks of cercarial dermatitis in new areas, cercarial dermatitis is considered as a reemerging disease [1]. Experimental infections of small mammals show that there might be a potential health risk for humans due to exposure to *T*. *regenti* cercariae (particularly for immunodeficient patients). If the parasite is not killed in the skin, then its neurotropic behavior might represent a serious problem. Unfortunately, no data on such human infections are available, and an appropriate diagnostic tool is still missing. Immunological or molecular methods for differential diagnosis of bird schistosomes are highly desirable for verification/exclusion of *T*. *regenti* as a causative agent of undetermined neurological disorders of animal and human patients.
